# Supplementary material for: Biased and unbiased estimation of the average length of stay in intensive care units in the Covid-19 pandemic
Source: Ann Intensive Care. 2020 Oct 16;10:135. doi: 10.1186/s13613-020-00749-6 (PMC7561433; doi:10.1186/s13613-020-00749-6)
Supplement: Supplementary file 1 — Additional file 1: Appendix S1. Simulation study. [file 13613_2020_749_MOESM1_ESM.docx]

**Supplementary Appendix 1: Simulation Study**

In this supplementary analysis, we aimed at comparing two estimation approaches on simulated datasets mimicking the expected lengths of stay in an ICU. The two approaches are those described in the article: *Discharged patients’ estimation* (DPE), defined as the mean of already discharged patients’ stays at the date of estimation, and *Censored patients’ estimation* (CPE) using data on all previously admitted patients, whether or not they are already discharged.

**Methods**

Data were generated as follows: admissions were simulated in a 30-bed ICU, thus mimicking the Zhongnan ICU studied in the article. In an emergent virus context, we considered that 30 patients were admitted on day 0 and other patients were admitted as soon as previous ones were discharged, so that 30 patients were hospitalized at any time.

in a first set of simulations, lengths of stay (LOS) were simulated by sampling with replacement from the 59 LOS observed in Zhongnan. The only missing LOS (59 days at the date of last follow-up) was imputed to 65 days. In a second set of simulation, LOS were drawn from a gamma distribution fitted to the whole set of observed data (shape: 1.82, rate: 0.0865). The means (95% prediction intervals) of simulated LOS were 21.0 (3.0, 65.0) and 21.0 (2.17, 60.7). Two datasets of 2,500 simulations were generated with these rules.

In order to estimate performances of average LOS (ALOS) estimation methods according to the accumulating data that become available over time, ALOS with their 95% confidence intervals (CI) were estimated every day from day 1 to day 125 with the DPE and CPE methods. The parametric distributions used for CPE were the exponential, gamma, Weibull and generalized gamma distributions.

Our primary judging criterion was the estimation bias, defined as the mean difference between estimated ALOS and theoretical value. Other criteria included the 95% CI coverage (proportion of 95% Cis covering the theoretical value) and the 95% CI width.

**Results**

Judgment criteria on estimation days 10, 20, 30, 60 and 90 following the first admissions are reported for methods DPE and CPE (gamma and Weibull distributions) in Table S1. Table S1 also documents the number of admitted and discharged patients available on each estimation day.

**Table S1. Performance of ALOS estimation on simulated data**

1. CPE and DPE performances in a 30-bed ICU that would be saturated with patients having LOS sampled in the distribution observed in the Zhongnan series

| **Day** | **N. adm** | **N. dis** |  | **DPE** | **CPE (gamma)** | **CPE (Weibull)** |
| --- | --- | --- | --- | --- | --- | --- |
| 10 | 38.5 | 8.5 | Estimate (95% CI) | 5.8 (4.0, 7.7) | 23.3 (12.6, 49.7) | 21.9 (11.6, 48.4) |
|  |  |  | Bias (days) | -15.2 | 2.3 | 0.9 |
|  |  |  | 95% CI coverage (%) | 0.0 | 88.3 | 82.8 |
| 20 | 52.2 | 22.2 | Estimate (95% CI) | 10.0 (8.0, 12.2) | 21.0 (15.4, 30.0) | 20.0 (14.9, 28.7) |
|  |  |  | Bias (days) | -11.0 | 0.0 | -0.9 |
|  |  |  | 95% CI coverage (%) | 0.0 | 92.8 | 87.1 |
| 30 | 66.9 | 36.9 | Estimate (95% CI) | 12.7 (10.5, 15.3) | 20.6 (16.3, 26.6) | 20.1 (16.0, 25.8) |
|  |  |  | Bias (days) | -8.3 | -0.4 | -0.9 |
|  |  |  | 95% CI coverage (%) | 0.0 | 90.8 | 86.9 |
| 60 | 109.9 | 79.9 | Estimate (95% CI) | 16.2 (14.0, 18.9) | 20.8 (17.7, 24.8) | 20.8 (17.7, 24.7) |
|  |  |  | Bias (days) | -4.8 | -0.2 | -0.2 |
|  |  |  | 95% CI coverage (%) | 8.2 | 92.2 | 91.2 |
| 90 | 152.5 | 122.5 | Estimate (95% CI) | 18.0 (15.9, 20.5) | 21.0 (18.3, 24.3) | 21.0 (18.4, 24.3) |
|  |  |  | Bias (days) | -3.0 | 0.0 | 0.0 |
|  |  |  | 95% CI coverage (%) | 31.6 | 92.4 | 92.6 |

1. CPE and DPE performances in a 30-bed ICU that would be saturated with patients having LOS sampled in a gamma distribution

| **Day** | **N. adm** | **N. dis** |  | **DPE** | **CPE (gamma)** | **CPE (Weibull)** |
| --- | --- | --- | --- | --- | --- | --- |
| 10 | 38.6 | 8.6 | Estimate (95% CI) | 5.8 (4.0, 7.7) | 23.8 (12.7, 52.9) | 22.5 (11.7, 54.2) |
|  |  |  | Bias (days) | -15.2 | 2.8 | 1.5 |
|  |  |  | 95% CI coverage (%) | 0.0 | 0.9 | 84.0 |
| 20 | 52 | 22 | Estimate (95% CI) | 9.9 (7.8, 12.3) | 21.5 (15.8, 31.1) | 20.4 (15.2, 29.8) |
|  |  |  | Bias (days) | -11.1 | 0.5 | -0.6 |
|  |  |  | 95% CI coverage (%) | 0.0 | 94.4 | 89.8 |
| 30 | 66.2 | 36.2 | Estimate (95% CI) | 12.7 (10.5, 15.3) | 21.3 (16.8, 27.4) | 20.6 (16.5, 26.4) |
|  |  |  | Bias (days) | -8.3 | 0.3 | -0.4 |
|  |  |  | 95% CI coverage (%) | 0.0 | 94.9 | 91.4 |
| 60 | 109.1 | 79.1 | Estimate (95% CI) | 16.7 (14.4, 19.4) | 21.1 (18.0, 24.9) | 20.9 (17.9, 24.6) |
|  |  |  | Bias (days) | -4.3 | 0.1 | -0.1 |
|  |  |  | 95% CI coverage (%) | 13.6 | 94.6 | 92.8 |
| 90 | 151.7 | 121.7 | Estimate (95% CI) | 18.2 (16.1, 20.7) | 21.1 (18.5, 24.1) | 21.0 (18.5, 24.0) |
|  |  |  | Bias (days) | -2.8 | 0.1 | 0.0 |
|  |  |  | 95% CI coverage (%) | 36.1 | 94.4 | 92.6 |

Performance of CPE (using either a gamma or Weibull distribution) and DPE are reported when estimation is made on day 10, 20, 30, 60 or 90 following the first admissions. For each day of estimation, the corresponding number of patients already admitted (N. adm) and discharged (N. dis) is shown. Judgement criteria are the mean ALOS point estimate and its 95% CI, the bias (mean difference between estimated ALOS and theoretical value) and the 95% confidence interval coverage.

Estimations from the first simulated dataset (sample of observed LOS at Zhongnan ICU) show that DPE expectedly underestimates the true ALOS at any time with a bias ranging from 15.2 days on day 10 to 3 days on day 90. CPE predictions were always more accurate, with a maximum bias of 2.3 and 0.9 days for the gamma and Weibull distributions, respectively. These methods show no bias for day 90 estimations. CPE with exponential and generalized gamma distributions are not shown as the former constantly provided worse estimates than the gamma and Weibull methods, whereas the latter was frequently unable to provide estimates on day 10 as the parametric model did not always converge. DPE 95% CI was unable to include the true ALOS up to day 30, with a 95% CI coverage reaching 31.6% on day 90. CPE methods performed better with 95% CI coverage of 88.3% and 82.8% on day 10 and 92.4% and 92.6% on day 90 for gamma and Weibull distributions, respectively.

Estimations from the second dataset (sample from a gamma distribution) gave very similar results. DPE underestimated ALOS while absolute bias was below 0.1 day for CPE estimates from day 60 on, both with a gamma and a Weibull fitting. The gamma distribution expectedly performed slightly better than the Weibull distribution to estimate LOS generated by a similar distribution, with a 95% CI coverage of 94.4% on day 20 vs. 89.8% for the Weibull distribution.

Evolution of DPE and CPE-based ALOS over time are detailed in Figure S1. In both datasets, CPE was unable to provide accurate estimates before day 10 approximately, with corresponding average numbers of admitted and discharged patients around 38.5 and 8.5, respectively. After such a delay, both CPE distributions gave accurate estimates, though they both tended to underestimate the true ALOS on the first dataset. On the second dataset, CPE with Weibull distribution slightly overestimated the true ALOS. We must also note that in the first dataset, about 60 days were needed before CPE perfectly estimated the true ALOS. As 4 out of the 59 observed stays last 59 days or more, such a long time is necessary for the model to account for the substantial frequency of very long stays, higher than what the gamma and Weibull distributions fitted on shorter stays would predict.

**Figure S2: Mean ALOS estimation along time from simulated data**


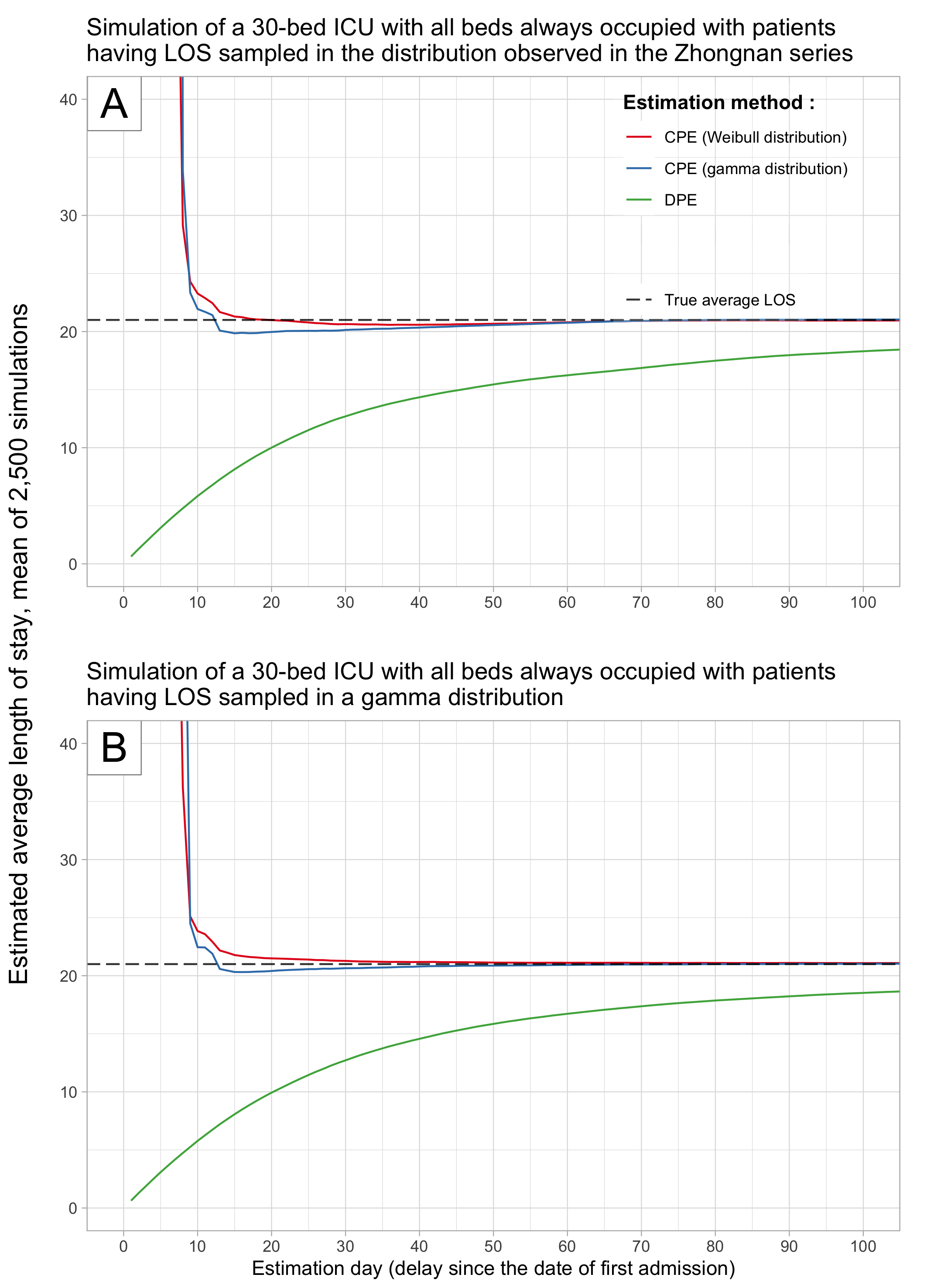


**Discussion**

These results show that in this setting, where no method provides accurate estimates in the first 10 days, then CPE estimates are constantly more accurate. Though CPE is presented as “unbiased”, we see that the reported bias is not null. Several observations can help explain these results:

First, CPE with the studied distributions is unbiased on a logarithmic scale, i.e. when estimating not the arithmetic mean but the geometric mean of LOS. An unbiased estimation, whose residuals are normally distributed around the true value, translates into a positive bias when exponentialized: this explains why CPE estimates always tend to be overestimated early in the follow-up. Actually, when plotting the geometric mean of estimated LOS rather than its arithmetic mean (data not shown), CPE methods underestimate LOS at the beginning of the simulation.

Second, we can wonder why, on the unbiased (logarithmic) scale, CPE underestimates ALOS. The main reason is that this method cannot provide any result when no patient is discharged as no information is available to estimate ALOS. Results reported during the first days of follow-up are therefore those from a subset of simulations with early discharges, thus creating a selection bias similar, to a lesser extent, to the one observed with DPE. Estimations made when only few discharges have occurred are still unreliable because of insufficient data for computing relevant confidence intervals.

For these reasons, we advise not to use CPE or any other estimation method before a sufficient number of patients are discharged. In our data, estimations become relevant after day 10 approximately, when at least 8 patients are discharged. As a rule of thumb based on sensitivity analyses, we suggest to apply this method when at least 5 patients are discharged and to carefully consider the 95% confidence interval rather than only the point estimate.

These simulations were based on a 30-bed ICU and results are not quantitatively transposable to other settings. The main difference between DPE and CPE relies on the consideration of admitted but not yet discharged patients, which is, in an overwhelmed healthcare system with maximal bed occupancy, the number of available beds. Therefore, though CPE should always be preferred to DPE, its benefits would be limited when applied to a ward with very few beds, as no method would be able to provide relevant estimates before a long time. On the other hand, applying CPE on data collected in a ward with a high number of beds, or by gathering information on stays from several wards could be very helpful for providing accurate estimations relatively early in the course of an outbreak.

To apply CPE, several parametric distributions are available. The exponential distribution relies on only one parameter, therefore fewer data are needed to fit a model than for distributions that require estimation of more parameters. However, we observed that this distribution is not flexible enough to fit data departing from this theoretical distribution; early estimations tend to importantly overestimate ALOS and we advise not to use this distribution. Weibull and gamma distributions are similar to the exponential with an additional parameter, therefore they are more flexible and provide significantly better fits. These distributions are both special cases of the generalized gamma distribution which relies on a third parameter. With a large amount of available data, the latter distribution will always provide better fits than the previous ones. However, when only few stays compose the available data set, estimation of the three parameters is delayed whereas Weibull and gamma distributions already provide relevant estimates and 95% CIs. Therefore, the generalized gamma distribution is likely not the best option for early estimates of ALOS and we advise to rather consider Weibull or gamma distributions for CPE.
