# Supplementary material for: Biased and unbiased estimation of the average length of stay in intensive care units in the Covid-19 pandemic
Source: Ann Intensive Care. 2020 Oct 16;10:135. doi: 10.1186/s13613-020-00749-6 (PMC7561433; doi:10.1186/s13613-020-00749-6)
Supplement: Supplementary file 2 — Additional file 2: Appendix S2. Unbiased average length of stay estimation—an illustrative example. [file 13613_2020_749_MOESM2_ESM.pdf]

Supplementary Appendix 2

Unbiased average length of stay estimation - An illustrative example

Contents

1 Cohort simulation **1**

2 ALOS estimation **4**

2.1 Overall estimation 5

2.1.1 Estimation from discharged patients 5

2.1.2 Gamma distribution 5

2.1.3 Weibull distribution 6

2.2 Multivariable modeling and consideration of co-variables 7

2.2.1 Estimation from discharged patients 7

2.2.2 Gamma distribution 7

2.2.3 Weibull distribution 9


```
# Packages installation / loading
if (!require(pacman)) install.packages("pacman")
```

```
## Loading required package: pacman
```

```
pacman::p_load(tidyverse, latex2exp, flexsurv, knitr)
```

1 Cohort simulation

This first part aims at simulating a template of cohort data to apply our estimation method If data is already available, you can skip this part.

Let us consider an hospital ward where patients are admitted on day day_{adm} and discharged on day day_{dis}. Patients can be admitted with a condition c_1 and/or a condition c_2. To simulate a cohort of 120 patients admitted between days 0 and 30, we will assume their lengths of stay (LOS) follows a Weibull distribution depending on presence of c_1 and c_2:

$$LOS_{c_1=0,c_2=0} \sim Weibull\left(\lambda = 12, k = 1.4\right)$$
$$LOS_{c_1=0,c_2=1} \sim Weibull\left(\lambda = 16, k = 1.4\right)$$
$$LOS_{c_1=1,c_2=0} \sim Weibull\left(\lambda = 18, k = 1.4\right)$$
$$LOS_{c_1=1,c_2=1} \sim Weibull\left(\lambda = 24, k = 1.4\right)$$

where λ and k are the scale and shape parameters, respectively.
These parameters are chosen so that the expected LOS depends on c_1 and c_2 with no interaction $\left(\frac{24}{12} = \frac{16}{12} \times \frac{18}{12}\right)$.

```
ggplot(data.frame(days = c(0, 50)), aes(days)) +
    stat_function(fun = function(x) dweibull(x, scale = 12, shape = 1.4),
                  aes(color = "No", linetype = "No")) +
```

```
  stat_function(fun = function(x) dweibull(x, scale = 16, shape = 1.4),
                aes(color = "No", linetype = "Yes")) +
  stat_function(fun = function(x) dweibull(x, scale = 18, shape = 1.4),
                aes(color = "Yes", linetype = "No")) +
  stat_function(fun = function(x) dweibull(x, scale = 24, shape = 1.4),
                aes(color = "Yes", linetype = "Yes")) +
scale_color_manual(TeX("Condition c_1"), values=c("cadetblue", "coral3")) +
scale_linetype_manual(TeX("Condition c_2"), values=c("dashed", "solid")) +
labs(title = TeX("Simulated lengths of stay depending on conditions c_1 and/or c_2"),
     y = "Density") +
coord_cartesian(x = c(0, 40)) +
theme_light() +
theme(legend.position = "bottom")
```

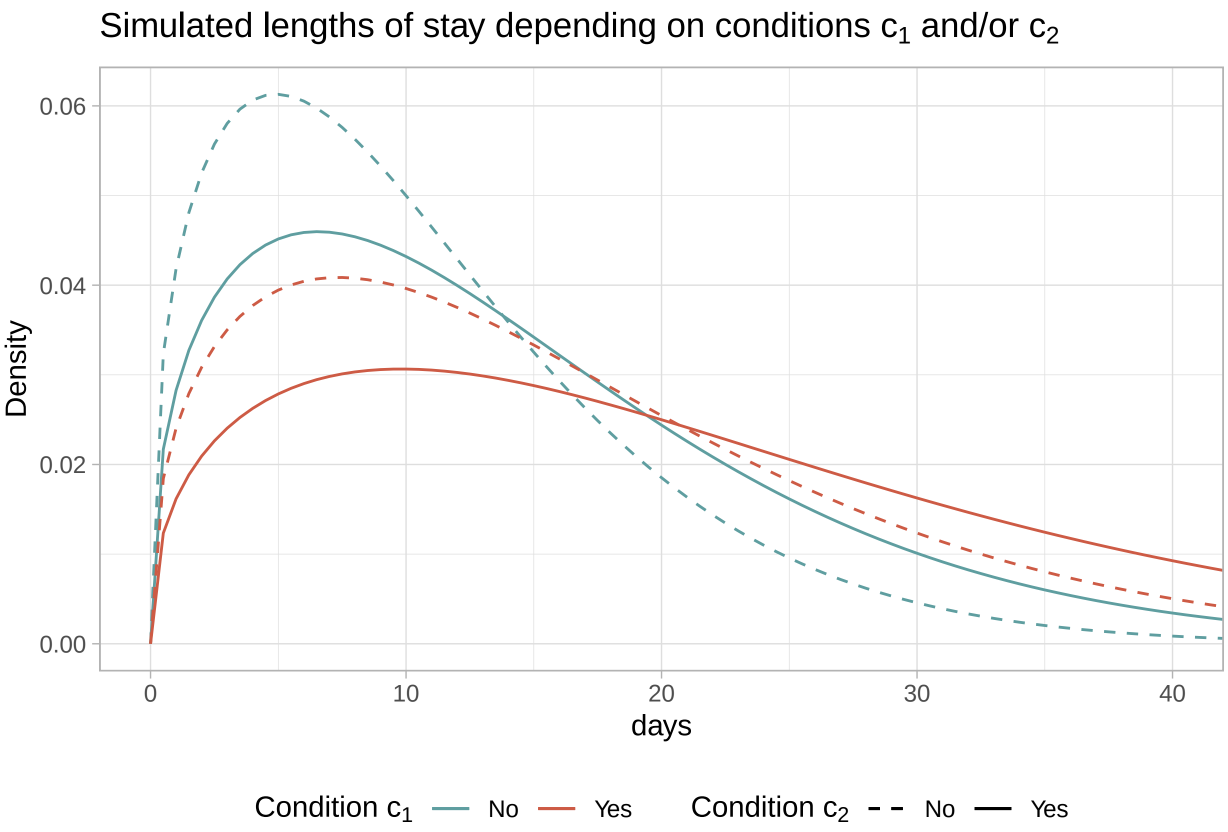

The expected LOS would then be:

$$E\left(LOS_{c_1=0,c_2=0}\right) = 10.94 \ days$$

$$E\left(LOS_{c_1=0,c_2=1}\right) = 14.58 \ days$$

$$E\left(LOS_{c_1=1,c_2=0}\right) = 16.41 \ days$$

$$E\left(LOS_{c_1=1,c_2=1}\right) = 21.87 \ days$$

Assuming 40% and 30% independent prevalences of Conditions c_1 and c_2, the overall expected length of stay would be:

$$E\left(LOS\right) = 16.75 \ days$$

```r
ggplot(data.frame(days = c(0, 50)), aes(days)) +
    stat_function(fun = function(x) dweibull(x, scale = 15.84, shape = 1.4),
                  color = "black", linetype = "solid") +
  labs(title = TeX("Simulated lengths of stay in the cohort"), y = "Density") +
  coord_cartesian(x = c(0, 40)) +
  theme_light()
```

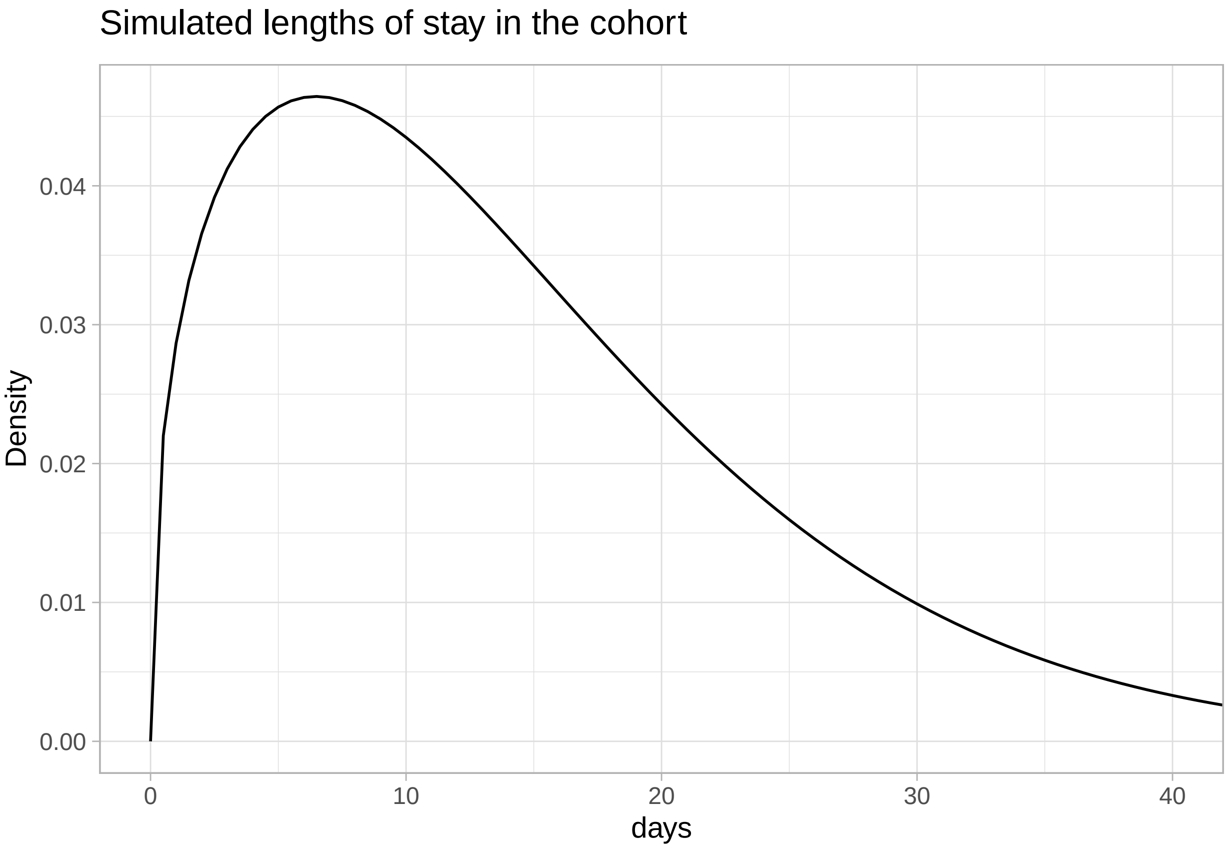

At the end of follow-up, collected data should look like these:

```r
n_patients <- 120

set.seed(123)

cohort <- tibble(
  c1 = rbinom(n_patients, 1, .4),
  c2 = rbinom(n_patients, 1, .3),
  day_adm = sample(0:30, n_patients, replace = TRUE),
  los = rweibull(n_patients, shape = 1.4,
                 scale = 12 ^ (!c1 & !c2) * 16 ^ (!c1 & c2) * 18 ^ (c1 & !c2) * 24 ^ (c1 & c2)),
  day_dis = day_adm + los
)
```

Sample of cohort data

```
head(cohort, 10) %>% kable()
```

c1	c2	day_adm	los	day_dis
0	0	28	2.463783	30.46378
1	0	13	20.225422	33.22542
0	0	20	12.107165	32.10716
1	0	15	6.560676	21.56068
1	0	22	25.663693	47.66369
0	1	28	43.302964	71.30296
0	0	0	11.129225	11.12923
1	0	7	14.339551	21.33955
0	0	7	10.802316	17.80232
0	0	9	12.599069	21.59907

where `c1` and `c2` describe the presence of conditions c_1 and c_2, `day_adm` the admission day, `los` the length of stay and `day_dis` the discharge day.

After the last patient is discharged (day 82 after the first admission), we can retrospectively estimate the average length of stay (ALOS):

```
mean(cohort$los)
```

```
## [1] 14.25218
```

And depending on c_1 and c_2 strata:

```
cohort %>%
  group_by(c1, c2) %>%
  summarise(n = n(), ALOS = mean(los)) %>%
  kable()
```

c1	c2	n	ALOS
0	0	52	11.57320
0	1	20	14.02546
1	0	39	16.41435
1	1	9	20.86515

Note that these estimates may be bad approximations of the true distribution of simulated LOS, especially in low-sample-size strata.

2 ALOS estimation

Now let us assume we need to estimate ALOS early in the follow-up, without waiting until the last patient is discharged (e.g. on day 25).

On this day, only a subset of patients is known and only some of them are discharged. Let us keep only patients admitted before day 25 and add:

- a dummy variable `discharged` when these patients are already discharged,
- a variable `obs_los` for the observed LOS, defined as the real LOS for discharged patients and the time since admission for patients not discharged yet.

```r
day_obs <- 25

cohort_sub <- cohort %>%
  filter(day_adm < day_obs) %>%
  mutate(
    discharged = ifelse(day_dis < day_obs, 1, 0),
    obs_los = ifelse(discharged == 1, los, day_obs - day_adm)
  )
```

2.1 Overall estimation

2.1.1 Estimation from discharged patients

A biased ALOS estimation would consider only discharged patients and underestimate the true ALOS:

```r
cohort_sub %>%
  filter(discharged == 1) %>%
  summarise(mean(los)) %>%
  kable()
```

mean(los)
8.750394

We advise to rather fit a parametric model accounting for censored data, using for example gamma or Weibull distributions (the generalized gamma distribution is even more flexible but requires a larger sample size to provide accurate estimates).

2.1.2 Gamma distribution

```r
fit_gamma <- flexsurvreg(Surv(obs_los, discharged) ~ 1, data = cohort_sub, dist = "gamma")
fit_gamma
```

```
## Call:
## flexsurvreg(formula = Surv(obs_los, discharged) ~ 1, data = cohort_sub,
##     dist = "gamma")
##
## Estimates:
##        est     L95%    U95%    se
## shape  1.6684  1.1836  2.3517  0.2922
## rate   0.1170  0.0716  0.1910  0.0293
##
## N = 90,  Events: 42,  Censored: 48
## Total time at risk: 714.5166
## Log-likelihood = -157.2152, df = 2
## AIC = 318.4304
```

This model allows us to estimate the ALOS and it's 95% confidence interval in the cohort:

```r
summary(fit_gamma, type = "mean", tidy = TRUE) %>%
  kable()
```

est	lcl	ucl
14.26194	11.22449	18.3244

Parametric estimates of the median LOS are also available:

```r
summary(fit_gamma, type = "quantile", quantiles = .5, tidy = TRUE) %>%
  kable()
```

quantile	est	lcl	ucl
0.5	11.53561	9.294747	14.49314

2.1.3 Weibull distribution

The Weibull distribution usually provides similar results.

```r
fit_weibull <- flexsurvreg(Surv(obs_los, discharged) ~ 1, data = cohort_sub, dist = "weibull")
fit_weibull
```

```
## Call:
## flexsurvreg(formula = Surv(obs_los, discharged) ~ 1, data = cohort_sub,
##     dist = "weibull")
##
## Estimates:
##        est     L95%    U95%    se
## shape   1.521   1.194   1.937   0.188
## scale  14.801  12.066  18.157   1.543
##
## N = 90,  Events: 42,  Censored: 48
## Total time at risk: 714.5166
## Log-likelihood = -156.1922, df = 2
## AIC = 316.3844
```

```r
summary(fit_weibull, type = "mean", tidy = TRUE) %>%
  kable()
```

est	lcl	ucl
13.34015	10.74001	16.7032

```r
summary(fit_weibull, type = "quantile", quantiles = .5, tidy = TRUE) %>%
  kable()
```

quantile	est	lcl	ucl
0.5	11.63182	9.578722	14.08484

2.2 Multivariable modeling and consideration of co-variables

Predictions may more explicitly consider co-factors in the model formula for various objectives such as documenting the variability of the estimate according to a given co-factor or providing estimates for different strata of the population. The following example illustrates a situation where estimates are provided in sub-population groups according to c_1 and c_2 values.

2.2.1 Estimation from discharged patients

In our example, estimating ALOS in discharged patients with conditions c_1 and/or c_2 would still provide unreliable estimates and we would have to wait even longer to reach a sample size large enough to get approximately correct estimates in all strata:

```
cohort_sub %>%
  filter(discharged == 1) %>%
  group_by(c1, c2) %>%
  summarise(mean(los)) %>%
  kable()
```

c1	c2	mean(los)
0	0	7.821280
0	1	8.188997
1	0	10.170456
1	1	11.479803

Parametric estimation of ALOS can account for available characteristics of patients as covariates in a mutivariable model.

2.2.2 Gamma distribution

```
fit_gamma2 <- flexsurvreg(Surv(obs_los, discharged) ~ c1 + c2, data = cohort_sub, dist = "gamma")
fit_gamma2
```

```
## Call:
## flexsurvreg(formula = Surv(obs_los, discharged) ~ c1 + c2, data = cohort_sub,
##     dist = "gamma")
##
## Estimates:
##        data mean  est      L95%     U95%     se       exp(est)  L95%
## shape       NA     1.7826   1.2606   2.5208   0.3151       NA        NA
## rate        NA     0.1664   0.1000   0.2768   0.0432       NA        NA
## c1      0.4111    -0.5196  -0.9737  -0.0655   0.2317   0.5948    0.3777
## c2      0.2333    -0.2337  -0.7544   0.2870   0.2657   0.7916    0.4703
##        U95%
```

```
## shape       NA
## rate        NA
## c1      0.9366
## c2      1.3324
##
## N = 90,  Events: 42,  Censored: 48
## Total time at risk: 714.5166
## Log-likelihood = -154.282, df = 4
## AIC = 316.564
```

Strata-specific predictions can be obtained from a table with expected patients characteristics:

```
new_patients <- tibble(
  c1 = c(0, 0, 1, 1),
  c2 = c(0, 1, 0, 1)
)


new_patients %>%
  kable()
```

c1	c2
0	0
0	1
1	0
1	1

```
summary(fit_gamma2, newdata = as.data.frame(new_patients), type = "mean", tidy = TRUE) %>%
  kable
```

est	lcl	ucl	c1	c2
10.71355	7.953683	14.11880	0	0
13.53374	8.220933	22.68118	0	1
18.01330	11.913356	26.52203	1	0
22.75506	13.165916	40.22275	1	1

```
summary(fit_gamma2, newdata = new_patients, type = "quantile", quantiles = .5, tidy = TRUE) %>%
  kable
```

quantile	est	lcl	ucl	c1	c2
0.5	8.790397	6.477792	11.61714	0	0
0.5	11.104349	6.920743	17.63102	0	1
0.5	14.779799	10.080274	21.72277	1	0
0.5	18.670380	10.884376	32.34239	1	1

2.2.3 Weibull distribution

```
fit_weibull2 <- flexsurvreg(Surv(obs_los, discharged) ~ c1 + c2, data = cohort_sub, dist = "weibull")
fit_weibull2
```

```
## Call:
## flexsurvreg(formula = Surv(obs_los, discharged) ~ c1 + c2, data = cohort_sub,
##     dist = "weibull")
##
## Estimates:
##        data mean  est      L95%     U95%     se       exp(est)  L95%
## shape       NA     1.6107   1.2624   2.0551   0.2002       NA        NA
## scale       NA    11.2938   8.7727  14.5395   1.4556       NA        NA
## c1      0.4111     0.4681   0.0656   0.8706   0.2054   1.5969    1.0678
## c2      0.2333     0.2542  -0.2036   0.7121   0.2336   1.2895    0.8158
##        U95%
## shape       NA
## scale       NA
## c1      2.3883
## c2      2.0382
##
## N = 90,  Events: 42,  Censored: 48
## Total time at risk: 714.5166
## Log-likelihood = -152.9372, df = 4
## AIC = 313.8744
```

```
summary(fit_weibull2, newdata = new_patients, type = "mean", tidy = TRUE) %>%
  kable
```

est	lcl	ucl	c1	c2
10.11962	7.952574	13.12852	0	0
13.04912	8.501720	20.43688	0	1
16.16021	11.557616	23.57332	1	0
20.83838	12.624316	35.45040	1	1

```
summary(fit_weibull2, newdata = new_patients, type = "quantile", quantiles = .5, tidy = TRUE) %>%
  kable
```

quantile	est	lcl	ucl	c1	c2
0.5	8.995397	6.915231	11.48800	0	0
0.5	11.599448	7.573293	17.83245	0	1
0.5	14.364919	10.341230	19.75012	1	0
0.5	18.523376	11.123776	28.64298	1	1
